# FGFR1 regulates proliferation and metastasis by targeting CCND1 in FGFR1 amplified lung cancer

**DOI:** 10.1080/19336918.2020.1766308

**Published:** 2020-05-18

**Authors:** Ying Yang, Tingting Lu, Ziming Li, Shun Lu

**Affiliations:** Shanghai Lung Cancer Center, Shanghai Chest Hospital, Shanghai Jiao Tong University, Shanghai, China

**Keywords:** CCND1, FGFR1, CO-IP, Epithelial-mesenchymal transition, lung cancer

## Abstract

**Aims**: The analysis of the online databases revealed that CCND1 expression is correlated with poor prognosis in LSCC. We aimed to explore the function of CCND1 in tumor progression in LSCC.

**Main methods**: The expression of mRNA was measured using qRT-PCR. Protein expression was assessed by Western blot. Cell migration and invasion were assessed by transwell assay.

**Key findings**: CCND1 was co-overexpressed with FGFR1 in lung cancer patients. Overexpression of CCND1 promoted LSCC cell proliferation and metastasis. FGFR1 promoted the processes of EMT through AKT/MAPK signaling by targeting CCND1 in FGFR1-amplification cell lines.

**Significance**: IIn conclusion, our study demonstrated the regulatory mechanism between CCND1 and FGFR1 in FGFR1 amplified LSCC. Co-targeting CCND1 and FGFR1 could provide greater clinical benefits.

## Introduction

Lung cancer is the leading cause of cancer related death worldwide with only 16.8% 5-year survival rate after diagnosis [1–4]. Targeted drugs have been developed in lung adenocarcinoma (LADC) for patients with driven gene positive patients such as epithelial growth factor receptor (EGFR) mutation and anaplastic lymphoma kinase (ALK) or ROS proto-oncogene 1(ROS1) rearrangements to inhibit tumor progression. However, the advance of targeted therapy in Lung squamous cell cancer (LSCC) is rare. Therefore, it is critical and urgent to identify the molecular mechanism underlying the occurrence and development of LSCC to explore the drug-able driver genes for the future strategies.

Fibroblast Growth Factor Receptor 1 (FGFR1) is a transmembrane protein and member of the fibroblast growth factor receptor family [5]. It is known that FGFR1 can promote tumor progression and invasion in various cancers including gastric cancer, prostate cancer, and breast cancer [6,7]. In previous studies, our group has demonstrated that FGFR1 interacted with Gli2, SOX2 and YAP to maintain stemness or EMT in FGFR1 amplified lung cancer, especially in LSCC [8–10]. FGFR1 amplification is the most common type in the FGFR1 dysfunction, with 20% in LSCC, 5–7% in SCLC, and 1–3% in LADC [11–14]. As a promising candidate in LSCC, small molecule drugs targeting FGFR1 were developed. BGJ398 and AZD4547 are selective inhibitors of FGFR 1–3 in phase Ib clinical trials [15,16]. However, the efficacy of these inhibitors are modest [17–19].

The D-type cyclins family members (D1, D2, and D3) along with its binding partners CDK 4/6 partially regulate G1 to S-phase transition of the cell cycle [20,21]. More than 35 distinct transcription factors are regulated by CCND1 expression [22]. The oncogenic roles of the CCND1 have been demonstrated in various studies, with numerous human cancers including thyroid breast, LADC, liver, colon, and prostate, overexpress CCND1 [23–27]. Notably, 1/3 of FGFR1-amplified tumors harbor amplification of CCND1 in breast cancer [28,29]. FGFR1 overexpression promotes resistance to CDK4/6 inhibitors. Moreover, CCND1 may mediate FGFR1-induced drug resistance in ER+ breast cancer [30,31]. However, few reports discussed the potential molecular mechanism between CCND1 and FGFR1 in FGFR amplified LSCC. Meanwhile, we found that higher expression of CCND1 predicted shorter OS in LSCC. Meanwhile, the expression of CCND1 wasn’t associated with OS in LADC. Therefore, as promising targets in LSCC, there is an urgent need to further explore the development and progression of CCND1 and FGFR1 in LSCC.

We hypothesis for the first time that CCND1 interacts with FGFR1 to promote EMT in LSCC in in vitro and in vivo. In this study, we observed that CCND1 and FGFR1 were co-overexpressed in LSCC.

## Materials and methods

### Cells and reagents

The human lung cancer cell lines (H1581 and HCC95) were obtained from the American Type Culture Collection. Both cell lines were identified by short tandem repeat (STR) profiling (Table.S1) [10,32]. The cell lines were cultured with Roswell Park Memorial Institute (RPMI 1640,HyClone) containing 10% fetal bovine serum (FBS, Gibco) in a humidified atmosphere about 37°C in 5% CO2. FGFR1 inhibitor AZD4547 was kindly provided by AstraZeneca Pharmaceutical Company [33].

### Plasmid construction and siRNA transfection

The CCND1 plasmid, siRNA and FGFR1 plasmid, siRNA were obtained from biolink (Shanghai, China). Plasmid and siRNA (50 nM) were transfected by Lipofectamine3000 kit (Invitrogen, Carlsbad, USA). The sequences of siRNA was described by us before [10].

### Colony formation assay

Cells were transfected into plasmid or siRNA. After 24 h, cells were digested by trypsin and then replanted at a density of 100 cells/1cm^2^. After incubation of 2–3 weeks, cells were fixed with methanol and stained with 0.1% crystal violet. Colonies with the diameter larger than 100 µm were counted, and each Colony formation assay was repeated in triple.

### Quantitative real-time PCR

Lung cancer tissues and non-tumor adjacent tissues (NATS) were obtained by Shanghai Chest Hospital, Jiao Tong University (Shanghai, China). Written consents approving the use of tissue samples for research purposes were provided from lung cancer patients. The study gained approval by the Institute Research Ethics Committee of Shanghai Jiao Tong University [9]. Total RNA was isolated from the above cells or frozen tissues using Trizol reagent (Invitrogen). The procedures was described by us before [9,10].The primer sequences of this study were as follows:

ZO-1 (forward 5´-AGCGAAGCCACCTGAAGATA-3´and reverse 5´-GATGGCCAGCAGGAATATGT-3´)

Snail (forward 5´- CGCGCTCTTTCCTCGTCAG −3´ and reverse 5´- TCCCAGATGAGCATTGGCAG −3´),

CCND1(forward 5ʹ-GGAGCCTATTCTGCCCATTT-3′)

(5ʹ-CGAGGTCATAGTTCCTGTTGGTG-3′reverse);

GAPDH (forward 5ʹ- AGAAGGCTGGGGCTCATTTG −3′)

(5ʹ- AGGGGCCATCCACAGTCTTC −3′reverse);

Data were calculated by using the comparative threshold cycle (Ct) method, and the results were expressed as fold difference normalized to GAPDH. The experiment was repeated three times. The procedure other primer sequences were described by us before [8,10].

### Transwell migration/invasion assay

Cells (3 × 104) with 1% FBS medium were seeded into an 8-μm pore membrane with or without Matrigel (CORNING, 356231, with the final concentration of 30%), and medium with 10% FBS was set in the lower chamber. After 24 hours incubation, cells on the upper layer were erased with a cotton swab. Cells on the lower layer were fixed in Methanol for 20 min and stained with crystal violet dye (0.1%) for 20 min. Cells were then counted at three randomly selected views for the subsequent calculations.

### CCK8 assay

CCK- 8 assay kit (Dojindo Laboratories, Kumamoto, Japan) was used to quantify the rate of proliferation under treatment. Briefly, 1–2*10^3^ cells were seeded into a 96-cell plate. After transfected with plasmid or siRNA r for 24 h, 10 μl CCK8 reagent was put into the medium per well. The absorbance (450 nm) was measured by the microplate reader (Synergy2, BioTek, Winooski, VT) after 1 to 3 hours co-incubation.

### Western blot and nuclear-cytoplasmic separation assay

Treated cells or tissues were lysed by RIPA with phosphatase inhibitor, protease inhibitor, and phenylmethylsulfonyl fluoride (PMSF).The procedure was described by us before [10]. All the antibodies were obtained from Cell Signaling Technology (Beverly, MA, USA). The subcellular protein was extracted by nuclear and cytoplasmic protein extraction kit (Thermo Scientific™78833/NE-PER™).

### Immunofluorescence microscopy

After being rehydrated, the tissues were incubated with primary antibody overnight. The secondary-antibody was donkey anti-rabbit IgG that conjugated with Alexa Fluor 594. Cell nuclei was stained with DAPI (Sigma). Cells were then photographed and then quantified by the immunofluorescence microscope (Leica DFC420C) [10,32].

### Immunohistochemistry (IHC)

After rehydrating, tissue specimens were incubated with primary antibodies for 1 hour, following by incubation with secondary antibodies. Diaminobenzidine-hydrogen peroxide (Sigma) was used as the chromogen. The counterstaining was conducted with 0.5% hematoxylin. The immune-histochemical grades are as follows: -, no staining; +, 10%, 10%, 50%, 50%, 50%, 50%, 50% -, no staining; +, 10%; ++, 10%–50%; and +++, >50%.

### In vivo xenograft assay and orthotropic lung tumor model

In the subcutaneous xenograft model, H1581 (1 × 10^6^ cells) in a volume of 50 µL was injected into the right side of the nude mouse. AZD4547 (12.5 mg/kg/d) or siRNA were given to nude mice by gavage or Intravenous injection for 3 weeks. In the orthotropic lung tumor model, cell suspension of H1581(2 × 10^6^ cells) in a total volume of 50 μL (Matrigel: PBS = 1:4) were injected directly into the left lung with insulin injection syringes (29 G*12.7 mm, BD, 328421). AZD4547 (12.5 mg/kg/d) was given by gavage for 2 weeks. All the nude mice were housed in the SPF (Specific Pathogen Free) animal room of Shanghai Jiao Tong University. All animal experiments were carried out in accordance with the approved scheme of the Shanghai Jiao Tong University Institutional Ethics Committee.

### Co-immunoprecipitation

Proteins extract were obtained from immunoprecipitation lysis buffer (P0013, Beyotime Biotechnology) with the Complete Protease Inhibitor Cocktail, and then centrifuged for 20 min (12,000 rpm,4°C). Soluble lysates were incubated with the antibodies at 4°C overnight, followed by incubation of Protein A/G Plus-Agarose(Santa Cruz Biotechnology, TX, USA) for 2 hr. Complexes were isolated from the beads and boiled for 10 minutes. Thermo Scientific Pierce Co-IP kit (Thermo Fisher Scientific) was used for the Co-IP assay. These obtained proteins were tested by Western blotting analysis.

### Analysis of public datasets from TCGA

Prognostic values of CCND1 level were downloaded from TCGA database and analyzed by Kaplan-Meier survival curves of NSCLC patients, using Kaplan-Meier Plotter (www.kmplot.com/analysis) [10,34].

### Statistical analysis

All the data were calculated as mean ± SEM or SD and analyzed using GraphPad Prism software (version7.0a, GraphPad Soft Inc.) [10]. All experiments are operated in triple. Student’s t tests was used to compare changes in mRNA levels between two groups. P value less than 0.05 was considered statistically significant.

## Results

### Upregulated CCND1 expression confers poor survival

**Figure 1.**
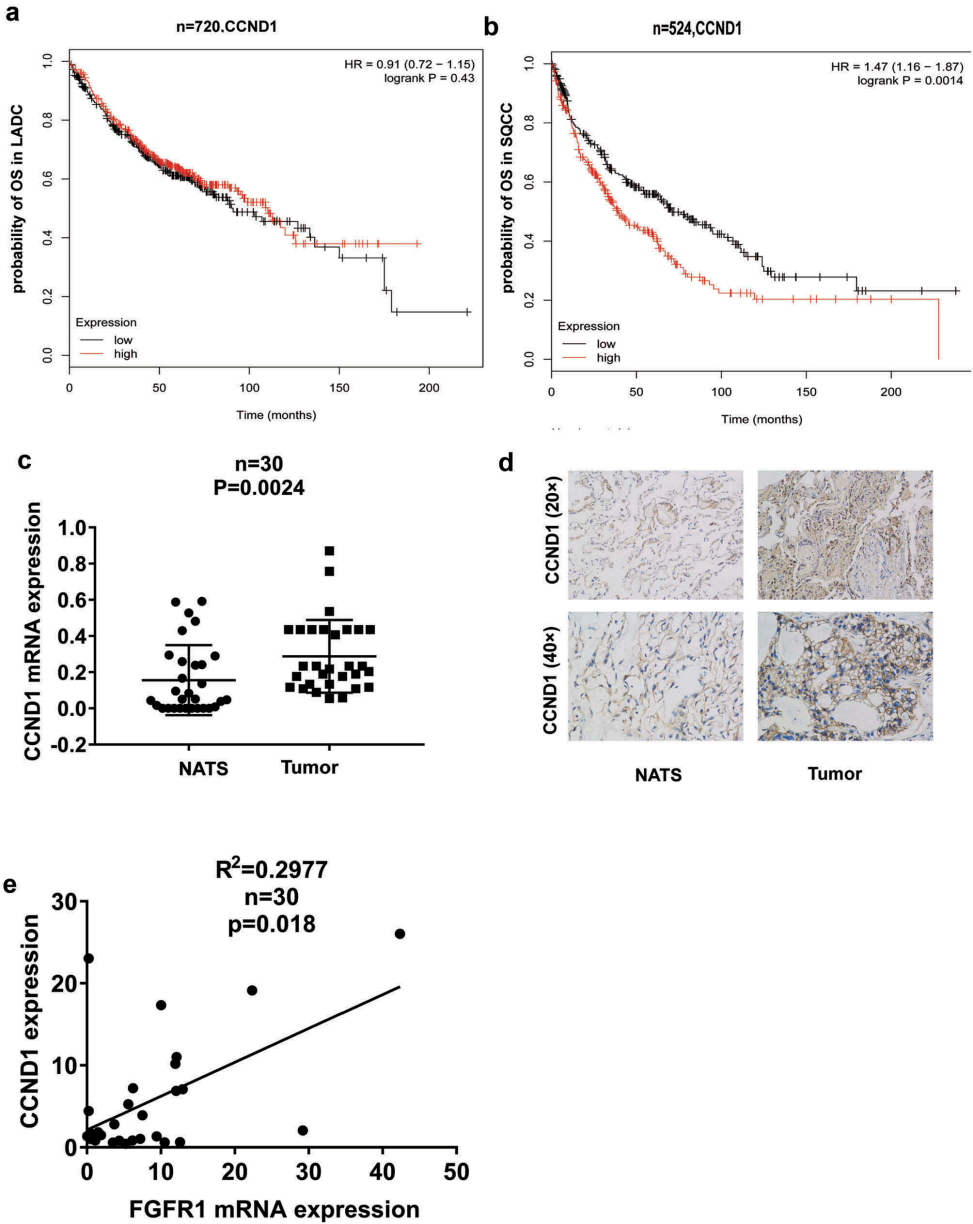
CCND1 deregulation correlated with survival rate. Kaplan–Meier curves depicting OS according to the expression of CCND1 in (a) LADC patients(n = 720) and (b) LSCC patients(n = 524) in TCGA database, (c)Relative CCND1 expression levels was detected by qRT-PCR in lung tumor tissues and non-tumor adjacent tissues(NATs), (d)The IHC staining of CCND1 in lung tumor tissues and non-tumor adjacent tissues(NATs), (e) Linear fit correlation analysis between FGFR1 mRNA and CCND1 were conducted in lung cancer patients(n = 30). P values were calculated by paired t-test. *p < 0.05;**p < 0.01; ***p < 0.001;****p < 0.0001.

We analyzed the relationship between CCND1 and clinical features. CCND1 was not associated with shorter OS in LADC (Figure 1(a)), but related to poor prognosis in LSCC (Figure 1(b)). According to quantitative RT-PCR data in 30 LSCC patients, we observed that CCND1 was overexpressed in tumor tissues compared to normal tissues (Figure 1(c)), which was further verified by immunohistochemistry analysis (Figure 1(d)). In addition, given to the previous result that FGFR1 is elevated in lung cancer [9,10]. We found a positive correlation between CCND1 and FGFR1 (Figure 1e).

Altogether, the above results suggested that both CCND1 and FGFR1 might involve in lung cancer progression.

### CCND1 promoted the proliferation, migration and invasion in FGFR1-amplified cells

**Figure 2.**
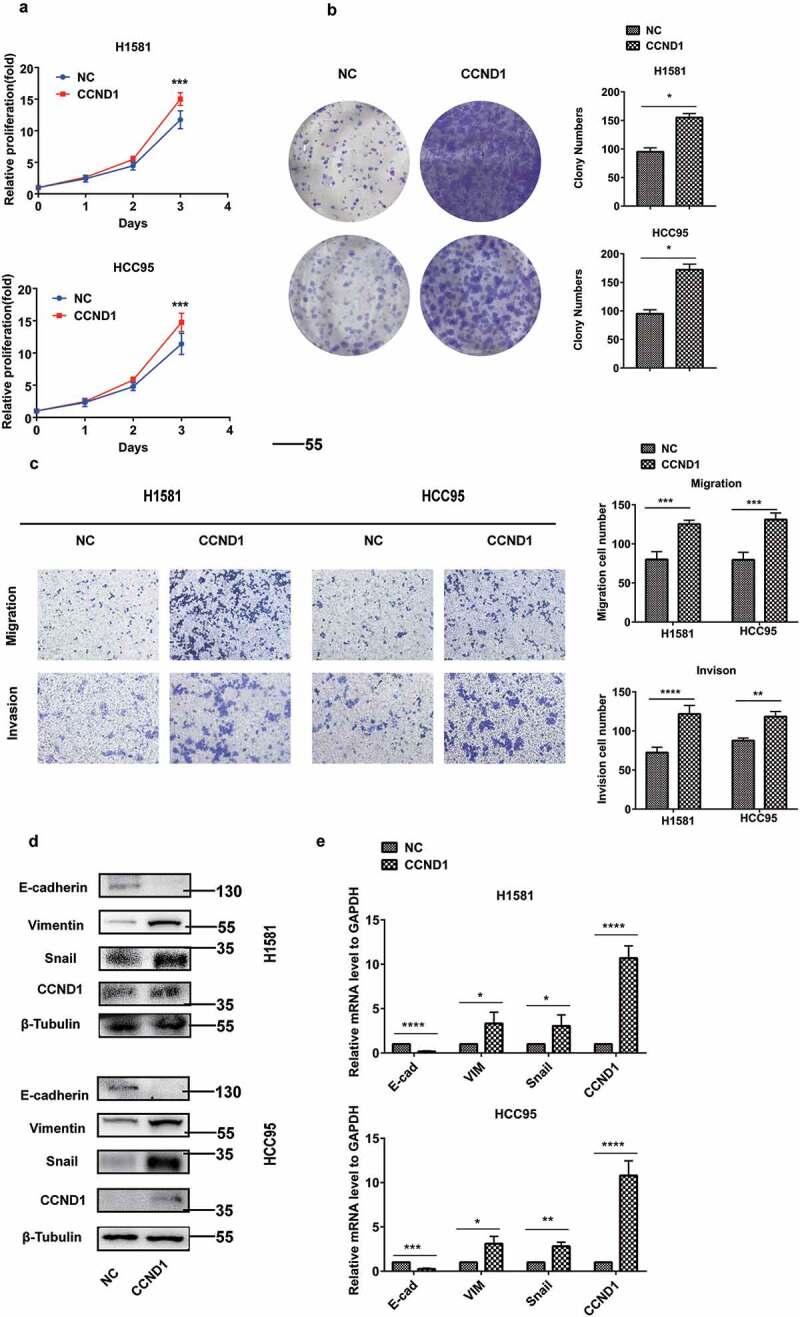
CCND1 promoted the proliferation, EMT process, and invasion in FGFR1-amplified lung cancer cell lines. H1581 and HCC95 cell lines were transfected with CCND1 overexpression plasmid. (a, b) Cell growth was measured by the CCK8 assay and clony assay, (c) Migration and invasion was determined by transwell assay, (d) Quantification of Represent EMT markers was measured by western blot, (e) Quantification of Represent EMT markers was measured by qRT-PCR. P values were calculated by Student t-test. * p < 0.05; ** p < 0.01; *** p < 0.001; **** p < 0.0001.

To explore the role of CCND1 in FGFR1-amplified lung cancer cells, H1581, and HCC95 cells as lung squamous cell carcinoma cell lines, with high expression of FGFR1, were used and authenticated by STR profiling (Table.S1). Both cells were transfected with CCND1 overexpression plasmid or si-CCND1. First, CCK8 assay and colony formation assay showed that CCND1 promoted the proliferation of H1581 and HCC95 cells (Figure 2(a) and (b)). In addition, CCND1 promoted migration and invasion as determined by transwell assay (Figure 2(c)). Moreover, CCND1 overexpression resulted in the upregulation of mesenchymal markers including vimentin (VIM) and snail, as well as the downregulation of epithelial markers such as E-cadherin (E-cad) and in both H1581 and HCC95 cells (Figure 2(d) and (e)).

**Figure 3.**
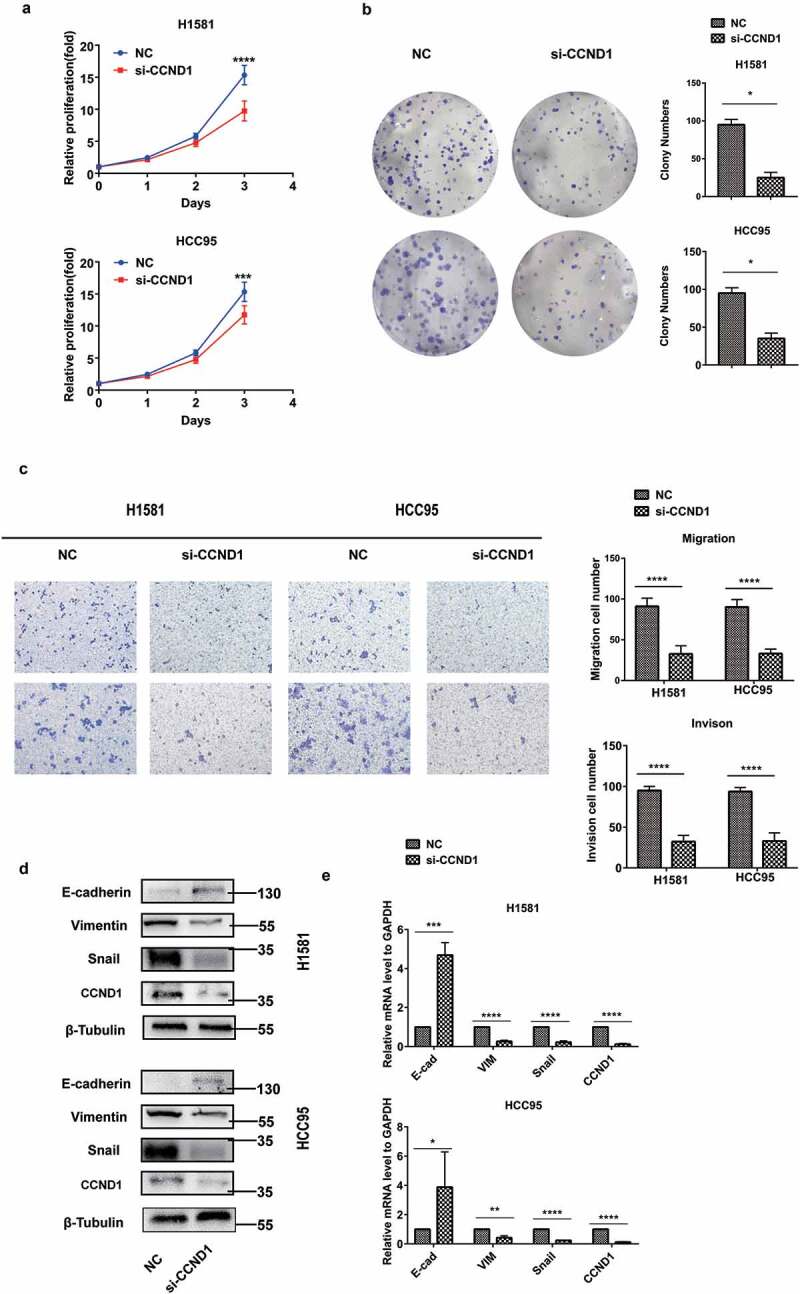
CCND1 inhibited the proliferation, EMT process, and invasion in FGFR1-amplified lung cancer cell lines. H1581 and HCC95 cell lines were transfected with si-CCND1. (a, b) Cell growth was measured by the CCK8 assay and clony assay, (c) Migration and invasion was determined by transwell assay, (d) Quantification of Represent EMT markers was measured by western blot, (e) Quantification of Represent EMT markers was measured by qRT-PCR. P values were calculated by Student t-test. * p < 0.05; ** p < 0.01; *** p < 0.001; **** p < 0.0001.

Conversely, CCND1 knockdown inhibited the proliferation, as determined by CCK8 assay and colony formation assay (Figure 3(a) and (b)). CCND1 knockdown inhibited the migration and invasion as determined by transwell assay (Figure 3(c)). CCND1 knockdown also sharply suppressed EMT process (Figure 3(d) and (e)).

These results implied that CCND1 promoted the EMT process in FGFR1-amplified lung cancer as an oncogene.

### CCND1 is a target of FGFR1

**Figure 4.**
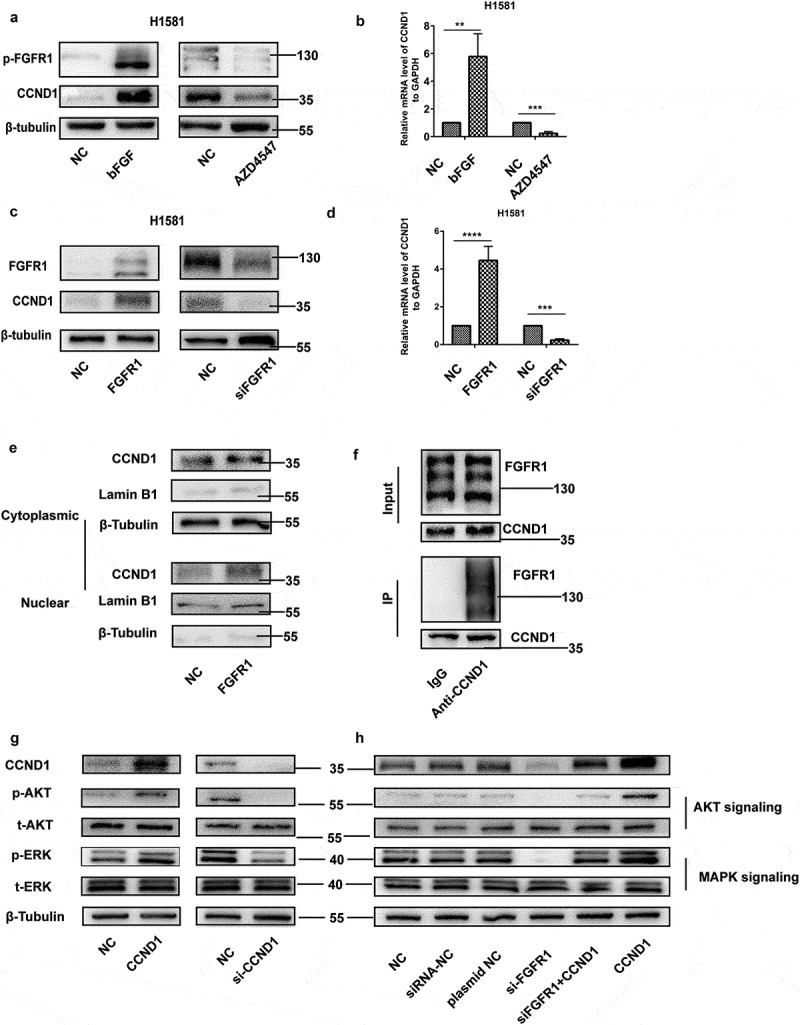
CCND1 is a target of FGFR1. (a) Western blot and (b)qRT-PCR analyzes of CCND1 expression levels in H1581 cells treated with FGFR1 ligand bFGF or FGFR1 inhibitor AZD4547, (c) Western blot and (d)qRT-PCR analyzes of CCND1 expression levels in H1581 cells transfected with FGFR1 overexpression plasmid or siRNA, (e) Western blot analyzes of CCND1 expression levels in cytoplasm and nucleus in H1581 cells transfected with FGFR1 overexpression plasmid, (f) CO-IP analyzes of FGFR1 expression levels after incubated with anti-CCND1 (g) After transfection of CCND1 overexpression plasmid or siRNA of CCND1 for 24 h, the protein levels of AKT and MAPK signaling pathways were measured by western blot. (h) After transfection of si-FGFR1 or CCND1 plasmid or co-transfection of si-FGFR1 and CCND1 plasmid for 24 h, the protein levels of AKT and MAPK signaling pathways were measured by western blot. P values were calculated by Student t-test. * p < 0.05; ** p < 0.01; *** p < 0.001; **** p < 0.0001.

CCND1 had a sharp downregulation after AZD4547 treatment(1 μM) and a significant upregulation after bFGF treatment(20 ng/mL) for 24 hours, which was detected by western blot (Figure 4(a)) and PCR (Figure 4(b)).To further explore the relationship between CCND1 and FGFR1, the overexpression plasmid and siRNA of FGFR1 were used. FGFR1 overexpress increased the expression of CCND1, while siRNA of FGFR1 decreased the expression of CCND1 (Figure 4(c) and (d)).To test whether the FGFR1 directly regulates CCND1, we conducted the following experiments. First, overexpression of FGFR1 promoted the migration of CCND1 from the cytoplasm into the nucleus (Figure 4(e)). In addition, Co-IP demonstrated that CCND1 bind to FGFR1 in physical structure (Figure 4(f)), which indicated CCND1 is a target of FGFR1. The downstream signaling pathway of FGFR1-CCND1 were explored. AKT and MAPK signaling, as the most motivation of EMT being reported, were upregulated by CCND1 overexpression plasmid and downregulated by si-CCND1 (Figure 4(g)). AKT and MAPK signaling was reported as the downstream signaling of FGFR1 [35]. The regulation of si-FGFR1 on AKT and MAPK signaling was abrogated by CCND1 overexpression plasmid (Figure 4(h)). In conclusion, we demonstrated that FGFR1 regulated the AKT/MAPK signaling by targeting CCND1.

### FGFR1 promotes proliferation, migration and invasion of NSCLC cells via targeting CCND1

**Figure 5.**
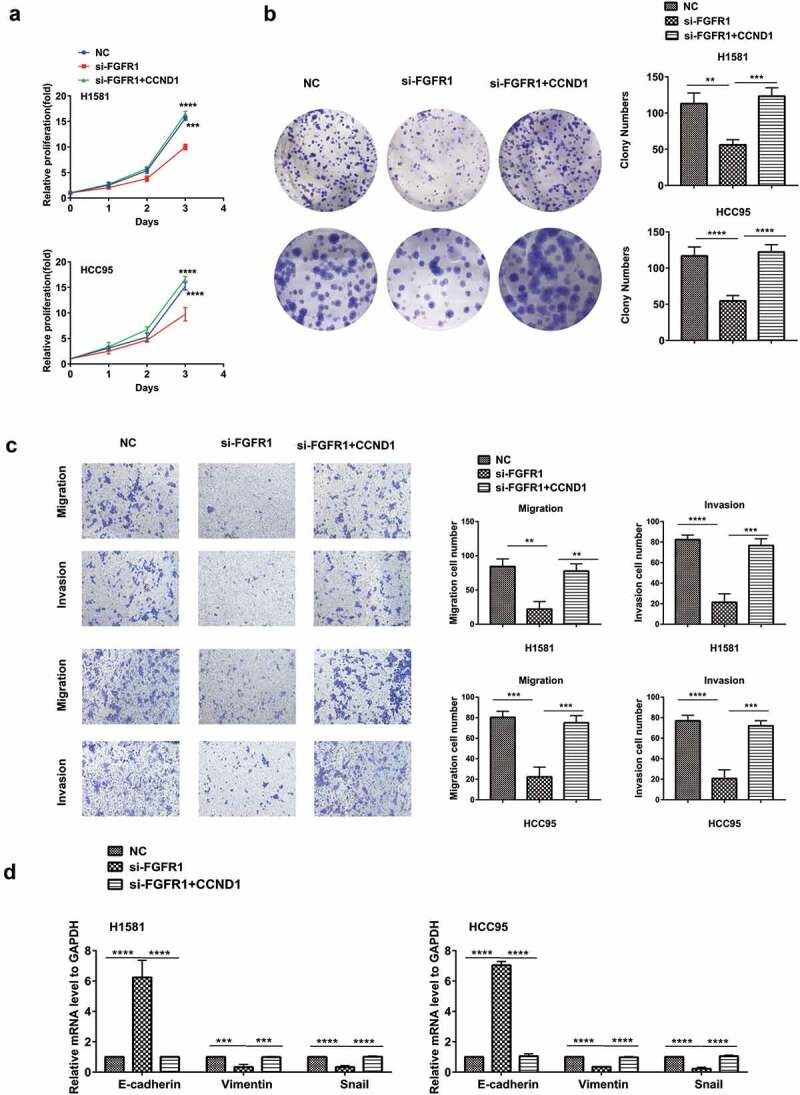
FGFR1 promotes proliferation, migration and invasion of NSCLC cells via targeting CCND1 H1581 and HCC95 cell lines were transfected with si-FGFR1 or siRNA-NC or co-transfection of si-FGFR1 and CCND1 overexpression plasmid. (a, b) Cell growth was measured by the CCK8 assay and clony assay. (c)Transwell assay was conducted to quantify the migration and invasion. (d) Quantification of Represent EMT markers was measured by PCR. P values were calculated by Student t-test. * p < 0.05; ** p < 0.01; *** p < 0.001; **** p < 0.0001.

To determine whether CCND1 acts a pivotal role in FGFR1-induced alterations in cell proliferation and metastasis, H1581 and HCC95 cells transfected with CCND1 overexpression plasmid and si-FGFR1 were used. The inhibitory effects of si-FGFR1 on proliferation (Figure 5(a) and (b)) and metastasis (Figure 5(c)) were abrogated by CCND1 overexpression plasmid in the cells. The down-regulation of mesenchymal markers including vimentin (VIM) and snail, and the upregulation of epithelial marker E-cadherin induced by si-FGFR1 were also rescued by CCND1 restoration (Figure 5(d)).

### Si-CCND1 and FGFR1 inhibitor exhibit synergistic antitumor effects in vitro and in vivo

**Figure 6.**
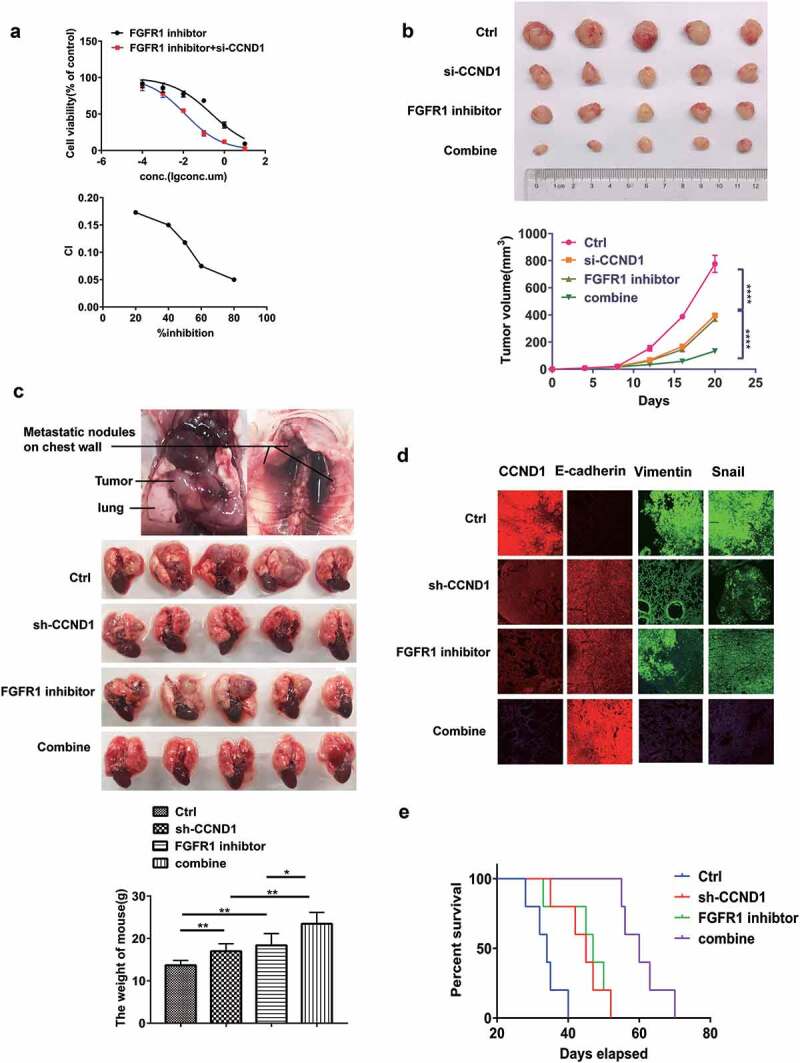
Si-CCND1 and FGFR1 inhibitor exhibited synergistic antitumor effects in vitro and in vivo. The experiments were conduct using H1581 cells (a) H1581 cells were transfected with 0.3 nM siRNA-NC or si-CCND1, incubating with AZD4547 in a serial dilution. After 72 hours, CCK8 was used to evaluate cellular proliferation. CI values were determined by non-linear regression methods at any given effect. (CI = 1, additivity; CI.1, antagonism; CI,1, synergy) (b) Representative images showing tumor formation in the nude mouse treated with siRNA-NC, AZD4547, si-CCND1 or si-CCND1 with AZD4547 for 3 weeks(combine).(C,D,E) H1581 (2 × 10^6^ cells) transfected with sh-CCND1 or LV-NC in a volume of 50 μL (PBS:Matrigel = 4:1) were injected into the left lung of 6-week-old male BCLB/C nude mice (n = 20). One week after injection, 5 mice injected with sh-CCND1 and 5mice injected with LV-NC were treated with FGFR1 inhibitor AZD4547 (12.5 mg/kg/d) randomly for 3 weeks. (c) Representative images showing tumor formation in the nude mice treated with shRNA-NC, AZD4547, sh-CCND1 or sh-CCND1 with AZD4547 for 3 weeks (d) Quantification of Represent EMT markers was measured by Immunofluorescence. (e) Survival curve for the mice in each treatment group evaluated. P values were calculated by Student t-test. * p < 0.05; ** p < 0.01; *** p < 0.001; **** p < 0.0001.

As the co-overexpression of CCND1 and FGFR1 in LSCC, we hypothesized that the co-target CCND1 and FGFR1 may provide higher efficiency. To verify this hypothesis, H1581 cells were treated with increasing concentration of AZD4547, ranging from 0.01–10 μM, in combination with si-CCND1 or siRNA-NC using at a weak, fixed concentration (0.3 nM). The IC50 for was 0.2433 μM and 11.81 μM for AZD4547 and si-CCND1, respectively. However, the IC50 for AZD4547 plus si-CCND1 was reduced to 0.0668 μM. To further explore the interaction of CCND1 and AZD4547, we calculated the CI index between them (CI = CA,x**/**ICx,A + CB,x**/**ICx,B). Combination index (CI) is widely used to evaluate the efficiency of combination between drugs [36,37]. CI values were determined by non-linear regression methods at any given effect. (CI = 1, additivity; CI>1, antagonism; CI<1, synergy). The CI values was 0.15 at IC50(CI<1), indicating a strong synergy between si-CCND1 and FGFR1 inhibitor AZD4547 (Figure 6(a)).

To further verify the synergism in vivo, subcutaneous mouse models and orthotopic lung cancer mouse models were established using H1581 (Figure 6(b)–(e)). After a week, siRNA or siRNA-NC was injected into the implanted tumor every 3 days for a total of seven injections in subcutaneous mouse models. AZD4547 (12.5 mg/kg/d) was given by gavage for 3 weeks. The control group was given by gavage with saline. Tumor volume was measured every 3 days. The results indicated the subcutaneous tumors in si-CCND1 group grew slower than the siRNA-NC group. Meanwhile, the si-CCND1 plus AZD4547 group grew even slower than the si-CCND1 group or the AZD4547 group (p < 0.05, Figure 6(b)).

The orthotopic lung cancer mouse models were established. The volume of primary lung cancer and the number of metastatic nodules both decreased in an orderly fashion for the following four groups: the NC group, the AZD4547 group, the sh-CCND1 group and the sh-CCND1 plus AZD4547 group (p < 0.05, Figure 6(c)–(e)). The sh-CCND1 group plus AZD4547 group had the longest survival time (Figure 6(e)). Immunofluorescence staining for the representative EMT markers were also applied (Figure 6(d)). In sh-CCND1 group plus AZD4547 group, FGFR1 and mesenchymal markers Snail and Vimentin were significantly suppressed, while the epithelial marker E-cadherin was highly expressed (Figure 6(d)).

Overall, the downregulation of CCND1 exhibited remarkable antitumor efficacy and developed the synergistic antitumor effects with FGFR1 inhibitor in vitro and in vivo, which provides strong evidence that CCND1 can serve not only as a prognostic marker, but also as a vital target in therapies against FGFR1-amplified lung cancer.

## Discussion

Prior studies that have noted the importance of CCND1 and FGFR dysfunction in the initiation of cancers [38–31]. In this study, we showed for the first time that CCND1 strongly modulates the EMT phenotypes of LSCC. Thus, this provides strong evidence that CCND1 can serve not only as a prognostic marker, but also as a useful target in therapies against FGFR1-amplified LSCC.

The molecular etiology of lung cancer is complex, with a diverse reorganization of gene expression patterns promoting stemness and suppressing differentiation. It has long been recognized that FGFRs are overexpressed in various types of tumor, including lung cancer, breast cancer and oral SQCC [14,40,41]. As mentioned above, FGFR1 as an attractive target demonstrated modest response [42]. Therefore, signaling paradigm co-alternation with FGFR1 or genes have been widely studied, such as the co-active receptor tyrosine kinases, the co-activation of MTOR pathway, et al. [15,19,43,44]. We demonstrated the co-expression of FGFR1 and CCND1 in lung cancer.

Previous studies suggest that FGFR1 could regulate MAPK/PI3K-AKT signaling [45]. We confirmed that CCND1 activated the activity of MAPK/PI3K-AKT signaling, and overexpression of CCND1 offs the this effect of FGFR1 on MAPK/PI3K-AKT signaling, which suggest that FGFR1 partial inhibited MAPK/PI3K-AKT signaling pathway by suppression of CCND1.

Meanwhile, FGFR1 amplification/overexpression was reported as a mechanism of resistance to treatment with CDK4/6 inhibitors in combination with antiestrogens [31]. Given that, we explored the efficiency of co-target CCND-1 and FGFR1. Here, we confirmed the antitumor synergism between si-CCND1 and FGFR1 inhibitor AZD4547 in vitro and in vivo. In the cell experiment, the CI index is about 0.15, showing a strong synergistic effect. Furthermore, subcutaneous and orthotopic lung cancer models also confirmed the synergistic effect. The mechanism of the synergistic effect remains unclear, and we found FGFR1 inhibitor AZD4547 sharply downregulated the level of CCND1. The inhibitory effect of AZD4547 and si-RNA on CCND1 might be synergistic, which need further exploration.

Despite current studies pointing to the fact that CCND1 is overexpressed and activated in a variety of cancers [46], few reports discuss the underlying molecular mechanisms by which CCND1 is regulated or activated in malignant tumors, especially in FGFR1 amplified lung cancer. Notably, our efforts focused on an in vivo orthotropic mouse model, which closely mimics the clinical features of human lung cancer and clarifies that CCND1 plays a pivotal role in the initiation and metastasis of lung cancer.

In this study, we found that both nuclear CCND1 and FGFR1 protein expression are correlated and unregulated in LSCC. Overexpression of FGFR1 has previously been linked to shorter progress free survival (PFS) of lung cancer, and separately [35,47–49]. Our current efforts provide a mechanistic relationship between these two independent observations. The above findings indicated that CCND1 interacts with FGFR1 to endow EMT properties in lung cancer.

## Conclusions

Collectively, we discovered a regulatory axis between CCND1 and FGFR1. Si-CCND1 proved significant antitumor effect in the genetic background of FGFR1 amplified lung cancer. In addition, we found a synergistic antitumor effect between si-CCND1 and FGFR1 inhibitor AZD4547 in vitro and in vivo. These findings may provide new insights for the prognosis and treatment of patients with FGFR1-amplified lung cancer.
